# Case Report: Intracranial hypertension occurring in association with subacute thyroiditis

**DOI:** 10.3389/fneur.2026.1938586

**Published:** 2026-09-17

**Authors:** Mariapia Griffo, Chiara Muzio, Elisabetta Bernardi, Matteo Azzimonti, Manuela Pintus, Laura Dell'Arti, Francesco Viola, Stefania Paola Corti

**Affiliations:** 1 Department of Pathophysiology and Transplantation (DEPT), Dino Ferrari Centre, Università degli Studi di Milano, Milan, Italy; 2 Ophthalmology Unit, Fondazione IRCCS Ca' Granda Ospedale Maggiore Policlinico, Milan, Italy; 3 Department of Clinical Sciences and Community Health, Università degli Studi di Milano, Milan, Italy; 4 Neurology Unit, Fondazione IRCCS Ca' Granda Ospedale Maggiore Policlinico, Milan, Italy

**Keywords:** acetazolamide, intracranial hypertension, papilledema, pseudotumor cerebri syndrome, subacute thyroiditis, thyrotoxicosis

## Abstract

Subacute thyroiditis (SAT) is a self-limited inflammatory thyroid disorder that typically produces a transient thyrotoxic phase. Intracranial hypertension (IH) is a rare but potentially sight-threatening complication of thyrotoxicosis, reported almost exclusively in hyperthyroidism, with only a single previous report linking it to SAT. We report the case of a 29-year-old woman who developed IH during the thyrotoxic phase of subacute thyroiditis, temporally related to dental manipulation. She presented with headache, nausea, vomiting, and fever, followed by a syncopal episode; clinical, laboratory, and imaging findings led to a diagnosis of subacute thyroiditis with overt thyrotoxicosis. She later developed bilateral visual blurring and pulsatile tinnitus, and ophthalmological evaluation showed bilateral papilledema with reduced visual acuity (20/100 right eye, 20/33 left eye) and visual field defects. Lumbar puncture demonstrated an opening pressure of 47.6 cmH_2_O (35 mmHg) with normal cerebrospinal fluid composition; neuroimaging did not document venous sinus thrombosis or structural lesions. She was started on acetazolamide and corticosteroids, with subsequent recovery of visual acuity, although an inferior visual field defect persisted in the right eye. This is the second reported case of IH associated with subacute thyroiditis. This case underscores that fundus examination should be performed in every patient with a new or unexplained headache, since papilledema may cause irreversible optic nerve damage before visual symptoms appear, and that early treatment with acetazolamide, together with management of the underlying thyroid disorder, can prevent permanent visual loss.

## Introduction

Subacute thyroiditis (SAT) is a self-limited inflammatory condition of the thyroid gland, most commonly caused by viral infection, that usually manifests with cervical pain, fever, malaise, and transient thyroid dysfunction (1). Typically, an initial thyrotoxic phase lasting weeks to months, caused by destruction of thyroid follicles and release of preformed thyroid hormone into the circulation, is followed by a transient hypothyroid phase (up to 2 months) due to hormone depletion, and eventually by a return to a euthyroid state (1).

Thyrotoxicosis is a broad term that encompasses any clinical state resulting from excess of thyroid hormone levels, regardless of the source, and must be differentiated from hyperthyroidism, which specifically denotes conditions characterized by increased *de novo* synthesis and secretion of thyroid hormone by the thyroid gland (e.g., Graves disease, toxic multinodular goiter) (2). In subacute thyroiditis, thyrotoxicosis results from inflammatory destruction of thyroid follicles and passive release of stored hormone rather than from autonomous thyroid hyperfunction; this implies that antithyroid drugs (thionamides) are ineffective in SAT, since there is no increased hormone synthesis. Treatment relies instead on symptomatic relief with beta-blockers for thyrotoxic symptoms and NSAIDs or corticosteroids for pain and inflammation (1, 2).

While thyrotoxicosis is primarily recognized for its cardiovascular, metabolic, and gastrointestinal manifestations, it can also produce a range of neurological complications. These include fine postural tremor, hyperreflexia, proximal myopathy (thyrotoxic myopathy), anxiety, irritability, insomnia, cognitive disturbances, seizures, peripheral neuropathy, and hypokalemic periodic paralysis (3, 4). Less commonly recognized, thyrotoxicosis has been associated with intracranial hypertension (IH), a rare but potentially sight-threatening complication (5–8).

Idiopathic intracranial hypertension (IIH) is defined by elevated intracranial pressure (opening pressure > 25 cmH_2_O; > 18.4 mmHG) with normal cerebrospinal fluid (CSF) composition, in the absence of any identifiable secondary cause, including intracranial mass lesions, hydrocephalus, or venous sinus thrombosis (9). IIH occurs predominantly in young, obese women of childbearing age, and its incidence has risen in recent decades in parallel with the global obesity epidemic (9–11). These features are recalled here only as a term of comparison; the present case is discussed throughout as a secondary pseudotumor cerebri syndrome rather than as IIH.

While IIH by definition lacks an identifiable cause, secondary forms of IH can arise from cerebral mass lesions, venous outflow abnormalities, medications (e.g., vitamin A derivatives, tetracyclines), endocrine disorders, and less commonly hypercapnia, anemia, renal failure, and Down and Turner syndromes. Among endocrine etiologies, thyroid dysfunction represents a recognized but underappreciated cause, with both hyper- and hypothyroidism, as well as abrupt hormonal shifts related to levothyroxine therapy, being implicated (5, 8, 12).

The association between thyrotoxicosis and IH, though rare, has been documented in approximately 10 case reports spanning over four decades. The earliest reports were by Dickman et al. (13) and Stern et al. (14) who described IH in the setting of Graves disease. Subsequent cases included a child with co-existing hypovitaminosis A and hyperthyroidism, (15) a young woman with Graves disease, (16) a child with reversible IH during hyperthyroidism, (17) and cases by Coutinho et al. (5) and Gutch et al. (12). Yaka and Cakmur (18) reported the only case attributable to a hyperfunctioning solitary thyroid nodule rather than Graves disease. In addition, a systematic review by Datta et al. (6) identified 21 cases of IH following levothyroxine replacement therapy, and IH has been described in the context of thyroid carcinoma (7).

Critically, all cases of IH associated with thyrotoxicosis reported up to 2021 involved either hyperthyroidism (predominantly Graves disease), in which thyroid hormone excess results from autonomous gland hyperfunction, or levothyroxine use (exogenous thyrotoxicosis). To date, only a single clinical case has linked IH with subacute thyroiditis, a condition in which thyrotoxicosis arises from inflammatory destruction and passive hormone release: Boonyatarp and colleagues (2021) reported a 36-year-old woman who developed IH during the thyrotoxic phase of SAT, with resolution following acetazolamide and normalization of thyroid function (8).

We report a second case of IH associated with subacute thyroiditis, temporally related to recent dental procedures, in which neurological and ophthalmological involvement proved essential for the diagnosis and management of IH, resulting in preservation of optic nerve function.

## Case presentation

A 29-year-old woman with no significant past medical history and a normal BMI (23.4 kg/m^2^) presented to the Emergency Department of another hospital 14 days after a dental procedure involving manipulation of a carious molar (day 0). She complained of cervical pain, low-grade fever, severe headache with nausea, vomiting, photophobia, and diarrhea, unresponsive to analgesics and antibiotics. Laboratory findings showed elevated inflammatory markers (CRP 137.67 mg/L), mild anemia (Hb 10.9 g/dL), tachycardia (HR 95–105 bpm), and low-grade fever (37.5 °C), with tenderness in the right temporal region and mildly hyperemic oral mucosa. Neurological examination was normal, head CT showed no abnormalities, and a diagnostic lumbar puncture yielded normal CSF (WBC 2/μL, protein 0.406 g/L, with negative viral panel, direct smear microscopy, and cultures); opening CSF pressure was not measured. She was discharged with a working diagnosis of migraine without aura exacerbated by a flu-like syndrome.

On day 22, she experienced a syncopal episode preceded by malaise, nausea, and tunnel vision while climbing stairs, with rapid and complete recovery, without confusion or focal neurological deficit. She presented to our Emergency Department and was admitted to the Internal Medicine Department for fever of unknown origin. A syncope workup (echocardiography, orthostatic testing) was unremarkable, and a comprehensive serologic screening for infections was negative. On Internal Medicine evaluation, a mildly tender thyroid swelling was identified. Thyroid function tests revealed overt thyrotoxicosis: suppressed TSH, FT3 8.8 ng/L, FT4 45.3 ng/L, with negative anti-TSH receptor and anti-TPO antibodies and anti-thyroglobulin antibodies just below the positivity threshold (55; positive > 60). Thyroid ultrasonography showed an enlarged, hypoechoic, and irregular thyroid with low vascularization. These findings were consistent with subacute (de Quervain) thyroiditis. She was treated with oral prednisone 50 mg daily and propranolol 20 mg twice daily and discharged.

On day 33 the patient complained of persistence of headache and developed transient episodes of blurred vision, accompanied by pulsatile tinnitus. Due to these symptoms, she re-presented to our Emergency Department on the same day, where ophthalmological assessment demonstrated visual acuity of 20/100 (2–3/10) in the right eye (RE) and 20/33 (6/10) in the left (LE), both not correctable. Fundoscopy revealed bilateral papilledema with blurred disc margins, more pronounced on the right (RE >> LE), without evidence of retinitis or vasculitis. Pupils were symmetric and reactive; ocular motility was full. Optical coherence tomography (OCT) showed reduced ganglion cell complex thickness, while retinal nerve fiber layer (RNFL) measurements were unreliable due to edema. Color fundus photography was not performed at this stage; ophthalmological monitoring relied on dilated fundoscopy, OCT, and Humphrey visual field testing. Humphrey 30–2 visual field testing revealed diffuse reduction in retinal sensitivity, predominantly in the inferior sectors of the RE, and initial, subtle inferonasal changes in the LE.

Neurological examination was unremarkable except for bilateral visual impairment (right eye more affected than left eye), fine postural hand tremor, and brisk deep tendon reflexes in the lower extremities, with bilateral unsustained ankle clonus. Notably, ocular movements were painless, with full extraocular motility and no diplopia.

Head CT and CT angiography of the head and neck excluded parenchymal and vascular abnormalities. The pituitary gland appeared thinned and flattened on the sellar floor, with increased visualization of CSF spaces (partial empty sella). The ventricular system was normal for age. Major dural venous sinuses were patent without thrombosis, with dominant right venous drainage.

She was immediately admitted to the Neurology Department and underwent a lumbar puncture that showed a CSF opening pressure of 47.6 cmH_2_O (35 mmHg), which decreased to 36.7 cmH_2_O (27 mmHg) after CSF withdrawal. CSF was clear and colorless, with WBC 2/μL, glucose 62 mg/dL, protein 19 mg/dL, and lactate 1.8 mmol/L. Multiplex PCR panel for meningitis was negative. Brain and orbital MRI with gadolinium confirmed bilateral posterior globe flattening, without optic nerve sheath distension. No signal abnormalities of the optic nerves, chiasm, or optic tracts were identified. Cerebellar tonsils were minimally ectopic at the foramen magnum. MR angiography and venography confirmed venous sinus asymmetry due to hypoplasia of the left transverse, sigmoid, and jugular sinus, all of which were patent with no evidence of venous thrombosis.

The left-sided caliber reduction uniformly involved the transverse, sigmoid, and jugular segments, without focal narrowing of the dominant right-sided sinuses or the abrupt caliber change typical of acquired stenosis, and was therefore interpreted as congenital hypoplasia. Additional etiological screening for alternative or contributory causes of pseudotumor cerebri syndrome was otherwise normal, and no concomitant autoimmune disorder was identified. The patient was taking no drug associated with intracranial hypertension; the antibiotic prescribed before the first emergency department visit was amoxicillin. Mild normocytic anemia (Hb 10.9 g/dL) and a markedly elevated CRP (137.67 mg/L) were present at first presentation.

Acetazolamide was initiated at 500 mg 3 times daily. Serial monitoring of thyroid function showed an initially suppressed TSH (< 0.005), with a progressive increase toward normal values and concurrent normalization of FT3 and FT4 levels. During hospitalization, headache and pulsatile tinnitus gradually improved. Ophthalmological re-evaluations showed progressive improvement of visual acuity (up to 5/10 in the RE and 9–10/10 in the LE) and visual field, along with significant reduction of papilledema. She was discharged in stable condition with de-escalation of therapy.

During the subsequent 6-month follow-up period, the ophthalmological course was favorable, although the episode did not resolve without sequelae; the neurological examination remained stable over time, with only occasional residual tension-type headaches. Visual acuity improved to 20/20 (10/10) bilaterally and papilledema resolved. An inferior visual field defect persisted in the RE, with OCT evidence of diffuse ganglion cell thinning, consistent with incomplete recovery of optic nerve damage from the initial episode of elevated intracranial pressure (Figure 1). Treatment was withdrawn 3 months after discharge. A timeline of the episode of care is summarized in Table 1.

**Table 1:** Timeline of the episode of care.

Timepoint	Clinical events, findings, and management
Day 0	Dental procedure with manipulation of a carious molar.
Day 14	Presentation to an outside emergency department with neck pain, low-grade fever, headache, nausea, vomiting, photophobia, and diarrhea. CRP 137.67 mg/L; mild anemia. Normal head CT and normal CSF, with opening pressure not measured. Discharged with a working diagnosis of migraine exacerbated by a flu-like syndrome.
Days 22–31	Syncopal episode with tunnel vision; admission to Internal Medicine for fever of unknown origin. Tender thyroid swelling; overt thyrotoxicosis (suppressed TSH, FT3 8.8 ng/L, FT4 45.3 ng/L). Thyroid ultrasonography consistent with subacute (de Quervain) thyroiditis. Prednisone 50 mg daily and propranolol 20 mg twice daily started.
Day 33	Persistent headache, transient blurred vision, and pulsatile tinnitus; re-presentation to the emergency department. Visual acuity 20/100 (right eye) and 20/33 (left eye); bilateral papilledema; reduced ganglion cell complex on OCT; visual field defects on Humphrey testing.
Day 33 Neurology admission	Lumbar puncture opening pressure 47.6 cmH2O (35 mmHg), decreasing to 36.7 cmH2O after CSF withdrawal; normal CSF composition. MRI showed partial empty sella and posterior globe flattening, with patent dural sinuses and hypoplasia of the left transverse, sigmoid, and jugular sinus. Acetazolamide 500 mg three times daily started.
Hospital course	Progressive normalization of thyroid function; improvement of headache, tinnitus, visual acuity, and papilledema. Discharge with de-escalation of therapy.
6-month follow-up	Visual acuity 20/20 bilaterally and resolution of papilledema. Persistent inferior visual field defect in the right eye with ganglion cell thinning on OCT. Treatment withdrawn three months after discharge. Persistence of tension-type headache with 7-8 episodes per month. NRS 5/10, absence of associated symptoms or exacerbating factors.

**Figure 1:**
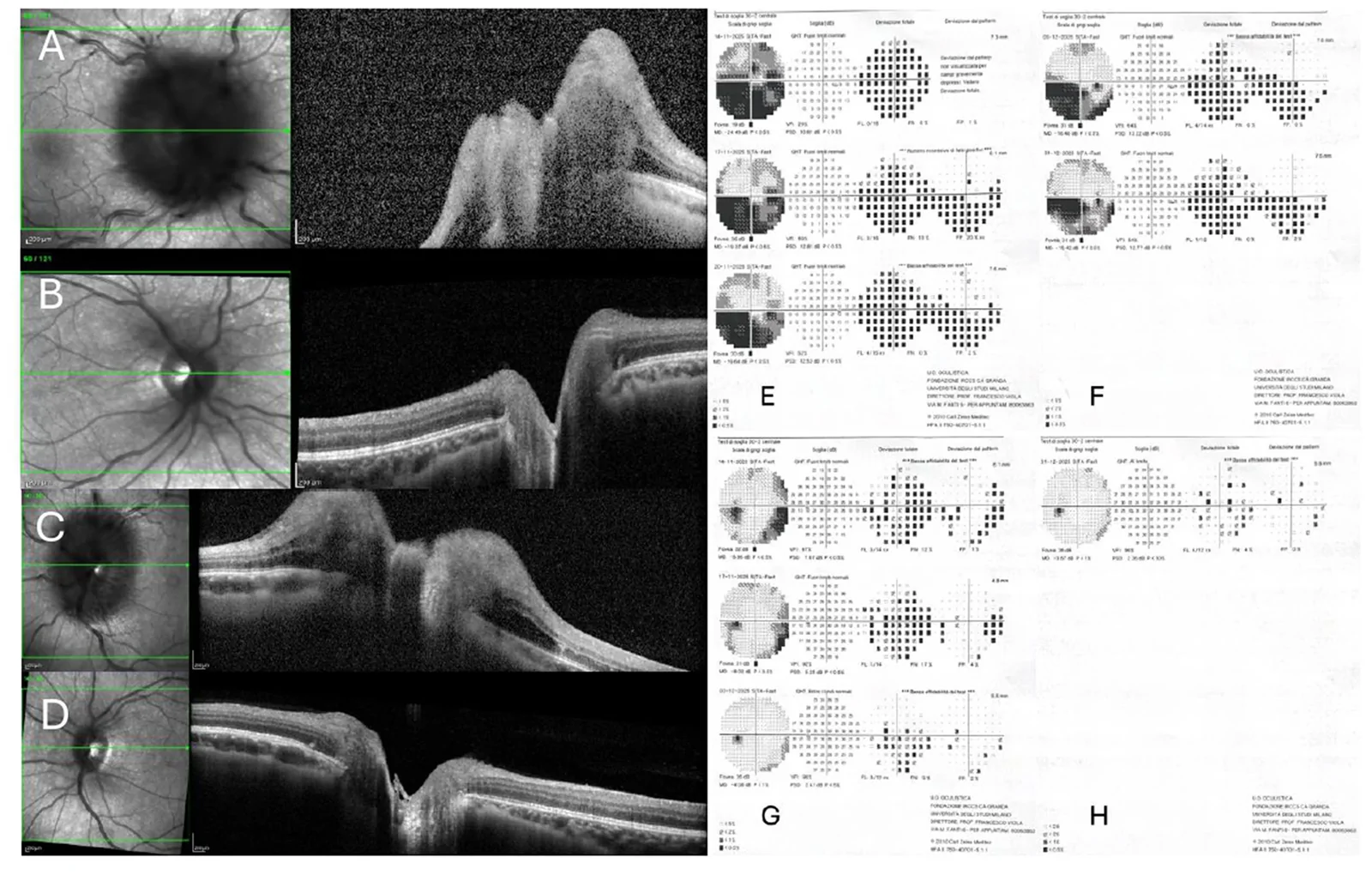
Multimodal ophthalmologic assessment, combining structural imaging (SD-OCT, **A–D**) and functional testing (Humphrey visual fields, **E–H**).

At the 6-month visit the patient was re-assessed systematically for symptoms that may persist after papilledema has resolved. Residual headache occurred 7–8 times per month with an intensity of NRS 5/10 and a tension-type phenotype, without pulsatile, positional, or Valsalva-related features. Pulsatile tinnitus completely resolved. She reported no dizziness, positional intolerance, imbalance, motion sensitivity, cognitive fogging, fatigue, and episodic disequilibrium. Symptoms were not influenced by exertion, posture, coughing, straining, the menstrual cycle, or sleep. She had returned to full-time work and to her previous level of physical exercise and she was able to drive. Body weight remained stable, thyroid function was normal off treatment, and OCT, visual fields, and neurological examination remained unchanged for 3 months after acetazolamide was discontinued, with no recurrence of papilledema.

## Discussion

The clinical presentation of our patient fulfilled the revised diagnostic criteria for definite pseudotumor cerebri syndrome (PTCS) proposed by Friedman et al. (21). Importantly, the Friedman criteria define PTCS as either primary (idiopathic intracranial hypertension, IIH) or secondary, when an identifiable cause is present. In our patient, the temporal association between overt thyrotoxicosis and the development of intracranial hypertension, in addition to the absence of the classic IIH risk factor (obesity), supported the interpretation of this case as PTCS associated with thyrotoxicosis in subacute thyroiditis.

The episode can be framed in three stages: an acute trigger (thyrotoxicosis with marked inflammation from subacute thyroiditis), a predisposing substrate (reduced venous outflow reserve from left transverse-sigmoid-jugular hypoplasia, concentrating drainage on a single right-sided route), and a dynamic response (increased arterial inflow, raised venous pressure, reduced CSF absorption gradient, and diminished craniospinal compliance). This remains a physiological hypothesis, not a demonstrated causal chain, as no venous or compliance variable was measured directly.

This case represents only the second reported instance of IH occurring during the thyrotoxic phase of subacute thyroiditis (8). Compared with the previously reported case, our patient presented with higher opening pressure (47.6 cmH_2_O), more severe papilledema, and worse visual outcomes, including a persistent inferior visual field defect with ganglion cell thinning on OCT. Our case emphasizes the potentially severe nature of this condition, highlighting the importance of early detection and timely intervention.

The pathophysiology of IH in the setting of thyrotoxicosis likely involves several interconnected mechanisms. Excess thyroid hormones reduce cerebral vascular resistance and increase cerebral blood flow, leading to elevated cerebral venous pressure (19, 20). Elevated venous pressure at the dural sinuses may impair the reabsorption of CSF through the arachnoid granulations, where a pressure gradient between the subarachnoid space and the venous system drives passive CSF drainage. The rise in venous pressure diminishes this gradient, potentially leading to CSF accumulation and intracranial hypertension (5, 19). In our patient, the congenital asymmetry of the venous drainage, with hypoplasia of the left transverse, sigmoid, and jugular sinus, may have reduced the venous outflow reserve and plausibly acted as a predisposing factor that amplified the rise in cerebral venous pressure. Another pathophysiological mechanism could involve electrolyte imbalances and extracellular water distribution due to thyroid hormone dysregulation (20). Moreover, thyroid hormones exert systemic effects by increasing cardiac output, thus contributing to hemodynamic changes, and by promoting a generalized inflammatory response that could alter blood-brain barrier permeability, with additional local inflammatory effects (3).

Increased cardiac output alone cannot explain the event: many physiological states, such as sustained aerobic exercise, raise cardiac output markedly without causing intracranial hypertension. Whether a hyperdynamic state raises intracranial pressure depends on factors beyond cardiac output, including cerebral autoregulation, venous outflow resistance, central venous pressure, craniospinal compliance, CSF secretion and absorption, collateral venous capacity, the speed of the hormonal change, inflammatory effects on the endothelium and blood-brain barrier, and individual anatomical susceptibility (24). In our patient, the combination of a rapid rise in thyroid hormone, a marked acute-phase response, and a single dominant venous outflow pathway plausibly narrowed the margin available to absorb an increase in cerebral blood volume.

Congenital venous hypoplasia, as seen here, must be distinguished from acquired transverse sinus stenosis: the former is a developmental variant reducing outflow reserve from the outset, whereas the latter often results from raised intracranial pressure and may reverse once pressure normalizes (24, 25). The venous asymmetry in our patient is therefore best regarded as a susceptibility factor rather than a demonstrated cause.

These considerations are better accommodated by a dynamic rather than a strictly volumetric reading of the Monro-Kellie doctrine. The classical fixed-volume model treats the three compartments equally and underestimates the venous side, even though cerebral blood inflow and outflow exceed CSF production by orders of magnitude; in reality, arterial and venous blood volumes, CSF displacement into the spinal canal, craniospinal compliance, venous outflow resistance, and cardiac pulsatility interact continuously (24). Consequently, a patient can have near-normal mean intracranial pressure yet abnormal pulse-pressure transmission, reduced compensatory reserve, or transient pressure rises under specific postural, respiratory, or hemodynamic conditions, which might in principle explain residual headache, pulsatile tinnitus, or dizziness after papilledema resolution. These remain untested physiological hypotheses, not findings in the present patient.

MR venography excluded sinus thrombosis but establishes only anatomical patency and cannot measure venous pressure or detect a trans-stenotic gradient. Catheter venography with manometry directly quantifies sinus pressures, identifies gradients of 8 mmHg or more, and characterizes jugular outflow limitation, and would have clarified whether the left-sided hypoplasia was a benign variant or a true reduction in outflow reserve (25, 26). Invasive investigation is not mandatory in every patient whose papilledema resolves; it is justified by persistent symptoms, recurrent pressure elevation, progressive visual findings, suspected venous obstruction, or consideration of venous sinus stenting (25). It was not performed in our patient because of the rapid resolution of papilledema and symptoms, which did not justify an invasive procedure.

Attributing the event to thyroid hormone excess alone would be an oversimplification. Subacute thyroiditis is an inflammatory condition, and the patient also had a markedly elevated CRP and mild normocytic anemia, a recognized contributor to pseudotumor cerebri syndrome (27). A multifactorial origin therefore seems more plausible, combining rapid hormonal change, systemic inflammation, altered fluid handling, and a constitutionally limited venous outflow reserve. Causality cannot be established from a single case, and these factors are presented as possible contributors rather than demonstrated mechanisms. No additional autoimmune disease or medication associated with intracranial hypertension was identified, and thrombophilia was not investigated.

The temporal proximity to dental manipulation involving a carious molar raises the possibility of an inflammatory or infectious trigger for subacute thyroiditis. SAT is commonly preceded by upper respiratory viral infections, and oropharyngeal procedures could plausibly trigger a systemic inflammatory response or facilitate viral dissemination (1). While a causal relationship cannot be established, the temporal sequence is noteworthy and merits consideration. Comprehensive infectious workup in our patient was negative, excluding bacteremia or direct infectious complications of the dental procedure.

The diagnostic workup in this case was complicated by the progressive, multisystem nature of the presentation. Initial symptoms were attributed to a flu-like syndrome; the subsequent syncopal episode and thyrotoxicosis shifted the diagnostic focus toward endocrine evaluation. It was only with the onset of visual symptoms and the fundoscopic finding of papilledema that IH was suspected, underscoring the importance of serial neurological and ophthalmological assessment in patients with thyrotoxicosis and evolving neurological complaints.

Notably, the absence of some classic neuroimaging markers of IIH (optic nerve sheath distension, transverse sinus stenosis), despite the presence of a partial empty sella and posterior globe flattening, in this patient highlights that clinical assessment and lumbar puncture with opening pressure measurement remain indispensable. Neuroimaging alone may not be sufficient to exclude IH when clinical suspicion is high (9).

The vast majority of described cases involve Graves disease, with only one prior case [Boonyatarp et al. (8)] attributed to subacute thyroiditis. Our case is therefore the second to document this specific association. Notably, the cases span a wide age range (childhood to 58 years) and include both sexes, suggesting that thyrotoxicosis-associated IH is not predominantly observed in young obese women, in contrast to IIH. A normal BMI (23.4 kg/m^2^) in our patient further supports this distinction and highlights the importance of thorough evaluation of secondary causes, particularly when typical risk factors for IIH are lacking.

Resolution of papilledema is an essential but partial outcome. A single lumbar puncture documents pressure at one moment and in one posture, and resolution of disc edema shows that the optic nerve head is no longer exposed to edema-producing pressure, but not that mean pressure, pulsatility, compliance, or venous outflow reserve have normalized under all conditions, a distinction relevant in patients with persistent symptoms after ophthalmological resolution (28). In our patient, the residual visual field defect and ganglion cell thinning establish permanent consequences; whether any abnormality of pressure physiology persisted is unanswered, as this would have required longitudinal assessment, serial OCT and perimetry, and, if justified, venous hemodynamic testing. We deliberately refrain from extending this reasoning to fibromyalgia, chronic pain syndromes, vestibular or cerebellar dysfunction, or other consequences for which we have neither symptoms, testing, nor supporting literature in this patient.

### Limitations

Several limitations should be taken into account. This is a single case report, which implies difficulty in drawing generalizable conclusions about the pathophysiology or optimal treatment approach. The temporal relationship between dental procedures and the onset of subacute thyroiditis, while suggestive, does not establish causality. Additionally, radioactive iodine uptake scintigraphy was not performed, although the clinical, laboratory, and ultrasonography findings were sufficient for SAT diagnosis. Fundus photography was likewise not obtained during the acute presentation, so photographic documentation of papilledema itself is not available; disc findings were instead followed serially by fundoscopy, OCT, and visual field testing. Future prospective studies and case registries would help clarify the incidence and mechanisms of this rare association. Cerebral venous pressure was not measured directly, so the contribution of the hypoplastic left venous pathway remains hypothetical. Craniospinal compliance and intracranial pressure pulsatility were not assessed by any invasive or non-invasive method, and intracranial pressure was documented at a single time point. Follow-up was limited to 6 months and was oriented primarily toward visual outcome, so subtle pressure-sensitive symptoms may have been under-ascertained.

## Conclusions

This case expands the very limited literature on IH associated with subacute thyroiditis and represents only the second such report. It demonstrates that IH in this context can present with severe papilledema resulting in persistent visual deficits if not promptly recognized and treated.

Headache is the most common symptom of pseudotumor cerebri syndrome, and papilledema may cause irreversible damage before visual symptoms appear, as in our patient, examined twice before fundoscopy. Fundus examination should therefore be performed in every patient with a new or unexplained headache, and suspected intracranial hypertension warrants prompt ophthalmological assessment, neuroimaging with venography, lumbar puncture, and early acetazolamide with treatment of the underlying thyroid disorder (22, 23). Resolution of papilledema is a necessary but not sufficient endpoint, and follow-up should also cover headache and other pressure-sensitive symptoms.

## Data Availability

The data supporting the conclusions of this article are individual clinical data and cannot be deposited in a public repository for reasons of patient confidentiality. Anonymized data will be made available by the authors, without undue reservation, on reasonable request.
